# Medical Insurance Representatives Perceptions on National Health Insurance Primary Healthcare Re-Engineering in South Africa: A Qualitative Study

**DOI:** 10.1177/21501319241237044

**Published:** 2024-04-03

**Authors:** Vivian Naidoo, Fatima Suleman, Varsha Bangalee

**Affiliations:** 1 University of KwaZulu-Natal, Durban, South Africa

**Keywords:** National Health Insurance, universal health coverage, South Africa

## Abstract

The South African government is moving toward universal health coverage (UHC) with the passing of the National Health Insurance (NHI) Bill. Access to quality primary healthcare (PHC) is the cornerstone of UHC principles. The South African governmental health department have begun focusing efforts on improving the efficiency and functionality of this system; that includes the involvement of private healthcare professionals and medical insurance companies. This study sought to explore perceptions of medical insurance company personnel on PHC re-engineering as part of NHI restructuring. A qualitative research design was adopted in this study. Semi-structured interviewed were conducted on 10 participants. Their responses were audio recorded and transcribed utilizing Microsoft Word^®^ documents. Nvivo^®^ was used to facilitate the analysis of data. A thematical approach was used to categories codes into themes. Although participants were in agreement with the current healthcare reform in South Africa. The findings of this study have highlighted several gaps in the NHI Bill at the current point in time. In order to achieve standardized quality of care at a primary level; it is imperative that reimbursement frameworks with clearly detailed service provision and accountability guidelines are developed.

## Background

The South African government has moved toward universal health coverage (UHC) with the passing of the National Health Insurance (NHI) Bill. The principle of UHC is to provide access to quality healthcare services to the population while ensuring financial protection of each citizen.^1^ The Bill seeks to correct the inequitable distribution of healthcare created by the current 2-tier healthcare system, that is, public and private sector.^2^ While the public sector is under significant stress and services between majority of the population; the private sector is largely funded through individual contributions, medical, and health insurance.^2^

Accessibility and affordability to quality primary healthcare (PHC) is a fundamental objective of UHC, but many healthcare systems around the world struggle to fulfil this basic healthcare need.^3^ The PHC system is intended to address the healthcare needs of all citizens at a community level and includes health promotion, rehabilitation, and disease prevention.^1^ To achieve UHC, the South African government has focused on re-engineering the PHC system to ensure equitable distribution of quality essential healthcare services.^2^

The NHI Bill details that healthcare services at each level of care will be provided by healthcare providers that are contracted and accredited by the NHI fund. A District Health Management Office will be established to facilitate, co-ordinate, and support the provision of PHC services at a district level.^2^ Contracting units for primary healthcare (CUPS) will be established to facilitate the provision of PHC services within a demarcated geographical area.^2^ The CUPS will be comprised of a community health center, district hospital, ward-based outreach teams, and private providers. There will be increased efforts in improving curative, rehabilitative, preventative, and health promotive services. Outreach teams will be supervised by a nursing team leader in each municipal ward to facilitate community visits.^2^

The NHI Fund, in consultation with the Minister of Health will determine the mechanism of payment to healthcare providers.^2^ For appropriate reimbursement, accredited providers must submit patient information on the Health Patient Registration System.^2^ Details related to PHC service packages and reimbursement of healthcare providers contracted by the fund have not been provided.

Private medical insurance companys’ role in the healthcare system will be targeted at providing cover for services not reimbursed within the NHI Fund.^4^ Medical insurance companies are of the opinion that this may be counterproductive as there are insufficient resources to provide full healthcare coverage to all citizens.^5^ This approach may subsequently increase the burden on the NHI system by draining resources that could be leveraged from the private sector.^5^

Given the proposed limitation to the role of medical insurance companies, it was necessary to gain an understanding on their perspectives of NHI PHC re-engineering and reimbursement.

The objectives of this study were to:

1. Explore medical insurance company representatives’ perspectives on NHI PHC re-engineering
2. To probe on potential mechanisms of reimbursement of healthcare providers from the perspective of these representatives.

## Methods

### Study Design and Population

A qualitative approach using semi-structured interviews was employed. A sample size of 10 pharmacist (Bachelor of pharmacy degree holders) that were employed at various medical insurance organizations in South Africa were selected via a snowball referral technique. By definition, a medical insurance organization provides financial protection for medical expenses for members that contribute monthly for a specific medical plan cover.^6^ For the purposes of confidentiality, the organizations from which participants were selected are not reported. Most of these organizations are concentrated in the KwaZulu-Natal, Gauteng, and Western Cape provinces in South Africa.

### Ethical Clearance

The University of KwaZulu-Natal Biomedical Research Ethics Committee, South Africa granted ethical clearance for this study (BREC Ref No. BE626/17). Participants were sent a formal invitation via electronic mail that detailed the aims and objectives of the study. Once verbal consent was obtained, participants were informed that their participation was voluntary and provided contact information of researchers. Participants were allocated a participation identification number to maintain anonymity throughout the study.

### Data Collection

Interviews were conducted in the English medium on an electronic online platform over a 5-month period, due to COVID-19 restrictions. Semi-structured interviews were conducted with 10 representatives that were selected via a snowball or referral sample technique. Semi-structured interviews have shown reliability and validity as a data collection tool in conducting qualitative research.^7^ All researchers have extensive expertise in conducting research of this nature. VCN has previous experience in conducting qualitative research utilizing semi-structured interviews and is a practicing pharmacist. VB and FS are senior researchers at the university and are registered pharmacist. The interview questions were developed by researchers collaboratively and designed to explore the perception of medical insurance representatives on NHI PHC re-engineering and reimbursement mechanisms. Open-ended questions were used to enable participants to express their perceptions and opinions explicitly. The draft questionnaire was piloted among 8 participants to prevent ambiguity and ensure questions were clear and concise. The final interview questionnaire comprised of 7 open-ended questions that discussed: NHI PHC re-engineering in South Africa and reimbursement mechanisms of healthcare providers. Probing was utilized during interviews to draw an in-depth understanding of the participants perception.

### Data Processing and Analysis

Interviews were recorded and transcribed verbatim utilizing Microsoft Word^®^. Transcript validation was conducted by 2 researchers. All researchers were actively aware that the line of questioning was in the capacity of researchers and not as healthcare professional. This enabled the interviewer to minimize further bias. Regular debriefings and review of field notes following interviews enabled researchers to reflect on observations. Analysis was initially conducted on 5 transcripts in which researchers noted that responses were uniform, therefore, a discontinuation criterion was reached with 5 additional transcripts. One researcher transcribed and coded all transcripts utilizing Nvivo^®^. Another researcher reviewed the preliminary codes and evaluated inconsistencies to avoid bias. The Braun and Clarke thematic analytical technique was utilized to consolidate the final codes and categorize them into themes.^8^ Predominant patterns identified in the data resulted in thematic identification and descriptions. To support the findings, verbatim quotations are reported in the results section.

## Results

### Sample characteristics

Ten medical insurance representatives were interviewed from various geographical locations across South Africa. Seven participants were female and 3 male with an average of 12 years of experience in the medical insurance field (Table 1).

**Table 1.** Participant Demographics.

Participant pseudonym	Gender	Geographical location	Years of experience in the medical insurance setting
Participant 1	Female	Gauteng	3
Participant 2	Female	Gauteng	3
Participant 3	Male	KwaZulu-Natal	7
Participant 4	Male	Gauteng	26
Participant 5	Female	Western Cape	20
Participant 6	Female	Western Cape	14
Participant 7	Female	KwaZulu-Natal	5
Participant 8	Female	KwaZulu-Natal	8
Participant 9	Male	Gauteng	18
Participant 10	Male	KwaZulu-Natal	11

The findings have been reported as 3 major themes that have been further expanded as sub-themes. The 3 major themes were: (1) Benefits of PHC re-engineering (Table 2); (2) Barriers with the reimbursement of healthcare providers (Table 3); and (3) Reimbursement mechanisms (Table 4).

**Table 2.** Occurrence of Themes and Sub-themes From Transcripts: Benefits of PHC Re-engineering.

Theme	Sub-theme	Total number of transcripts	Number of times in transcripts
Benefits of PHC re-engineering	Access to healthcare	7	11
	System efficiencies	4	8
	Enhanced referral pathway	3	4

Total number of transcripts: number of transcripts the theme was identified in.

Number of times in transcripts: Number of times the theme was mentioned by participant.

**Table 3.** Occurrence of Themes and Sub-Themes From Transcripts: Barriers to Reimbursement of Healthcare Professionals.

Theme	Sub-theme	Total number of transcripts	Number of times in transcripts
Barriers to reimbursement of healthcare professionals	Reimbursement system functionality	4	13
	Lack of information	9	14
	Lack of trust	7	7

Total number of transcripts: Number of transcripts the theme was identified in.

Number of times in transcripts: Number of times the theme was mentioned by participant.

**Table 4.** Occurrence of Themes and Sub-themes From Transcripts: Reimbursement Mechanisms.

Theme	Sub-theme	Total number of transcripts	Number of times in transcripts
Reimbursement mechanisms	Capitation	5	8
	Fee for service	5	7
	Performance/outcome based	2	2

Total number of transcripts: Number of transcripts the theme was identified in.

Number of times in transcripts: Number of times the theme was mentioned by participant.

#### Benefits of PHC re-engineering

##### Access to care

Participants expressed that the primary benefit of PHC re-engineering was enhanced access to essential healthcare services in communities.

*“I think it would be a good initiative. I think it would be good for the people of the community if services like that are available to them.” [Participant 8]*

*“I would love to see that expanded [referring to expansion of the service package] to be more inclusive and to give patients treatment options, rather than saying sorry this is the only thing that’s available for you.” [Participant 1]*

Participants felt that this would enable patients to be better managed at a PHC level and in turn reduce the severity of disease progression or presentation (Table 2).

*“There are certain conditions that we can manage for them to not become life threatening. I feel it needs to be re-engineered to allow more people to be able to help manage and lift the burden. .” [Participant 2]*

##### System efficiencies

Another key benefit expressed by participants is that PHC re-engineering could standardize service delivery received by South Africa citizens at a PHC level by adhering to standard treatment guidelines.

*“So in terms of the service provision, I think, as long as that access is the same and we following the standard treatment guidelines and essential medicines list.” [Participant 4]*

This participant further elaborated that this approach could result in significant cost savings as it promotes efficiency in the system.

*“As you move towards a multi-disciplinary and a multi professional practice, you’d be able to structure the health system much better. You’re bringing efficiency in delivery and can reduce the cost of delivering care. And therefore the reduced cost to the overall health system; in delivering care.” [Participant 4]*

##### Enhanced referral pathway

There was a general consensus that participants supported the governments endeavors to re-engineer the PHC system as it promoted continuation of care and enhanced the referral pathway.

*“Well I think it’s important for any health system that is being restructured to prioritise primary health care, not in isolation to tertiary healthcare, or, like hospital services, because everything is supposed to be connected, but they are supposed to be a strong referral mechanism that helps people to access higher levels of care. Primary Health Care is a backbone of any health system.” [Participant 10]*

*“I’m fully supportive of it. To me, to be able to deliver on care, you need to have nominated practitioners where that becomes the first point of call. You need to have multi-disciplinary team to look at all aspects of people’s health. From first of engagement, right through to recovery and rehabilitation.” [Participant 4]*

#### Barriers to reimbursement of healthcare professionals

##### Reimbursement system functionality

Participants identified that the current system does not promote functionality and enable healthcare professionals to charge for services rendered within the medical insurance scheme database; therefore, there is a need for electronic health record systems that allow healthcare providers to charge for services and be reimbursement under the NHI Fund.

*“The schemes do pay for the tariffs and we’ve had to pay for a few of them ourselves. The problem that comes in is that whatever systems the pharmacy is using like the CKS (Computer Kit Software) and things aren’t set up to charge tariffs, so they can’t submit a tariff code as a claim for the medical scheme to pay and everyone else blames the schemes, but they need to update their systems.” [Participant 1]*

*“It’s like you’re not sitting down and thinking policies through and you’re now creating all these structures, and then expecting policies to fit into structures. Instead it’s supposed to be structures that fit in policy, like business processes and computer systems. You don’t design computer systems, and then expect business processes to align to the computer systems, you develop computer business processes and then you see how the computer systems can aid the business process and create efficiencies.” [Participant 4]*

Additionally, participants expressed concern over the complexity of the system in order to appropriately claim for the service rendered.

*Well, it’s going to depend on how complex or simple the government’s claim system is going to be. There’s different levels. I don’t think that our system is going to be sophisticated enough to deal with claiming properly as to how much of work is going into each procedure that you’re doing. [Participant 3]*

One participant highlighted that the system should be designed to detect and minimize duplication of reimbursement submissions by healthcare professionals.

*“The biggest risk was service duplication. So you see a pharmacist, and then you see a doctor, and you have the whole thing duplicated, and we were not equipped, from an administrative perspective to block that or manage it correctly.” [Participant 9]*

##### Lack of information

A re-occurring sub-theme observed among participants was the lack of information and knowledge on how healthcare professionals will be reimbursed under the NHI fund.

*“Well, I don’t know. I’m trying to think about it, I’m trying to think about how that would actually happen, because you would provide a service but then I really don’t know how you would be reimbursed to be honest.” [Participant 26]*

*“I haven’t kept up to speed with that. I don’t even know if any of those details are in place and what the levels of reimbursement would be, and how to commensurate that with the current reimbursement.” [Participant 5]*

##### Lack of trust

It was noted that participants did not trust that the government would appropriately reimburse healthcare professionals for services rendered under the NHI Fund.

*“They charge a tariff code and then submitted to medical aid. I envision would be something similar but maybe at a lower cost but yeah you can’t really trust the government to pay; so I think that’s, you know, a big question mark over everybody’s heads.” [Participant 1]*

There was also concern that this system would be subject to corruption and mismanagement by enabling healthcare providers to manipulate the system.

*“That seems like it would be very open to corruption because that person is then also choosing the providers; and then providers have an incentive to be on that system.” [Participant 3]*

With that said, another participant expressed that although corruption is a major concern there are preventative measures in place to counteract this mismanagement.

*“The NHI will have a Fraud Unit. People have no confidence in that unit, and their concerns are noted and they are quite important concerns. The President has actually established a Fraud Unit, which is not sitting in the health sector, but sitting with a special investigations unit. There is a collaboration between the special investigations unit, and different stakeholders in the private sector, in the public sector, including the Auditor General. Remember, these contracting units will still be audited by the auditor general who is not part of the health sector and the special investigations unit should there be an issue of fraud that we’ll have to investigate.” [Participant 10]*

#### Lack of reimbursement mechanisms

Participants were probed on their perceptions and knowledge on the reimbursement mechanisms that could be utilized to reimburse healthcare providers for services provided at a PHC level.

##### Capitation

A large majority of participants were of the view that a capitation based approach was the most suitable. This approach reimburses providers based on the population that the provider is servicing by taking into account necessary epidemiological factors.

*“So if you think about it, a risk based capitation model. It looks at the risk and say, epidemiological risk of the catchment population that is assigned to that particular provider in that area, do they have more burden of disease, which burden of disease is more prominent across which age profile, and gender profile. And from that risk, then your risk adjusted capitation tariff will be determined which will cascade, up to a budget that will be allocated to that particular facility. Most medical schemes do use risk based capitation models for their primary health care services so it wouldn’t be much of an issue unless your question is the fund, having the technical capability to assess the risk that is submitted by different health delivery points.” [Participant 10]*

One participant expressed that in order to ensure that this system is efficient; services need to be costed appropriately to maintain the full benefit to the patient and remain profitable to the providers.

*“When the capitation level is not set correctly, it will without a doubt, compromise care because additional care comes with additional cost. So that is something you always have to think about with capitation, if you cost it badly. People either lose money, or stop providing services. And if you don’t, unpack your capitation experience and output to adjust the capitation fee correctly. Again, it’s going to be more a waste, than it is a service to the patient.” [Participant 9]*

##### Fee-for-service

Many participants felt that a fee-for-service mechanism of reimbursement was suitable particularly in a community pharmacy setting for services provided.

*“Should we need to create this tariff for different kinds of consultations for the pharmacist, and that goes back to the actual law and say, what is our scope, then create tariffs to say yes X patient came to the pharmacy and I gave this kind of service, and there’s a tariff for it, so that basically I see it. I see it working the same way as you know how doctors consultations are paid and many other providers paid, we created tariffs for the service.” [Participant 2]*

##### Performance/outcome based mechanisms

A small number of participants eluded to utilizing a performance based mechanism that takes into account that quality of service before reimbursing healthcare providers.

*“Performance based reimbursement is paying for value. So you need a scope of practice. You need a coding system for their scope of practice. You need remuneration system, but you need data collection on the access and the quality.” [Participant 4]*

With that said, another participant had doubts about the ability to quantify outcomes to a suitable reimbursement value.

*“Outcomes based reimbursement is wonderful terminology. I’d love to see somebody, measure it accurately. Being data obsessive, I can tell you how difficult it is to measure.” [Participant 9]*

## Discussion

An efficient PHC system is critical in attaining the objectives of UHC, which aims to improve accessibility to essential healthcare services.^1^ To achieve this, NHI PHC re-engineering efforts are focused on strengthening the system to ensure equitable distribution of high-quality healthcare services within communities. Participants supported the governments initiatives in transforming the PHC system because of enhanced accessibility. Studies from other countries further support these findings that an efficient PHC system is envisioned to promote accessibility and availability of healthcare services which produce positive population health outcomes.^9-11^

Participants highlighted that the quality of care received at a primary level must be standardized and regulated. Regulation of healthcare service packages enables the government to control the quality and safety of care offered by healthcare providers.^12^ Standardized healthcare packages can be beneficial in directing resource allocation to priority areas of service delivery.^12^ Delivering standardized PHC can be challenging as clinical frameworks are generally based on evidence from high income countries (HICs) that may ignore the resource constraints experienced by LMICs.^13^ Findings from a study that focused on standardizing PHC service delivery in a South African setting found that there is a pressing need to align clinical care with healthcare policies.^13^ Discrepancies and gaps in healthcare policies had direct effects on practice on the ground and resource availability. Clarifying healthcare provider roles and scope of practice ensured that providers fulfil their responsibilities and strengthened the relationship between staff at different levels of care.^13^ Given the critical impact the functionality of the PHC system has on the entire healthcare systems, there is merit in participants concern on the standardization of care.

The lack of trust in government to reimburse providers is another concern expressed by participants. The primary reason is the risk of corruption and mismanagement of funds for private gain. Corruptive practices in South Africa has been a longstanding problem that has hindered the accessibility and availability to essential services and resources.^14^ Ranking a bleak 32 on the Corruption Perceptions Index in the Sub-Saharan African region; the high prevalence of corruption is due to the lack of accountability and stringent regulatory frameworks.^14,15^ There is validity in participant concerns as it has been previously observed that countries with high levels of corruption spent less on healthcare.^16^ This resulted in an increased rate of poor health-related outcomes such as lower population life expectancy, immunization rates, and higher infant and child mortality rates.^16^ Other studies have shown that corruption affects the more vulnerable populations resulting in challenges in accessing essential healthcare services and higher out-of-pocket health associated expenses.^16^ International anti-corruption strategies have focused on strengthening accountability and enforcement, enhanced community engagement, improved transparency, and increasing remunerative scales of healthcare providers.^16^ These strategies should be incorporated into NHI processes to reduce potential corruption and promote transparency in the healthcare system.

In order to achieve the objectives of PHC re-engineering it is imperative that healthcare providers are reimbursed appropriately for services rendered. Participants expressed the need for a suitable electronic health record systems that is integrated with a reimbursement system. Currently, the lack of an appropriate system in the South African healthcare system poses a risk in delivering quality healthcare services and ensuring financial sustainability of providers. The implementation of such a system could promote billing practices and support the reimbursement of providers for services rendered under the NHI Fund. In a study on the utilization of electronic health record systems, it was found that there was improved quality of care as patient related information was easily accessible amongst the healthcare team thereby reducing treatment duplication.^17^ There was also a notable financial benefit as there was a reduction in medicine related expenses, billing errors, and improved capturing of charges for healthcare services by providers.^17^ However, while these technological advancements offer efficiency and functionality improvements, caution is warranted. There is the risk of misalignment of UHC principles as metropolitan areas may have greater digital access as opposed to rural areas, resulting in a disparity in service delivery operational processes.^18^

Another concern observed amongst participants is the lack of information especially on the reimbursement of healthcare providers. Information sharing is crucial in the successful implementation of healthcare policies. For successful healthcare system reform at a national magnitude, role-players would need to be informed in order to implement sound policies and ensure quality of care. This in turn will strengthen performance accountability amongst providers. Healthcare systems generally focus on services and facility outputs, and not on individual service experiences between patients and providers. Therefore, health provider performance needs to be linked to financial accountability in that the financial resources should account for the goods, services, and positive outcomes of each patient.^19^ Provider payment should be maximized on quality of care, efficiency, patient satisfaction, and equity. However, performance accountability is dependent on policy accountability as the criteria for performance and service delivery needs to be clearly stipulated.^19^ Without the necessary information, participants felt that not enough attention is being given to clear reimbursement models that account for patient outcomes, which was perceived as a gap in the current Bill.

The role of medical insurance companies with NHI implementation remains elusive, South Africa’s largest medical insurance provider stated that they may continue coverage while clarity on the NHI system is being sought.^5^ The reasons being (1) there is uncertainty as to when NHI will be fully implemented and functional; (2) services to be covered by NHI have not been clearly outlined and standardized; and (3) the Bill states that medical aid insurance cannot cover services that can be reimbursed under the NHI Fund and patients will have to follow the referral pathway for reimbursement of providers, but these services and pathways have not been defined.^5^ This gap in the referral process requires extensive consideration as it could hinder the functionality of the PHC system. Furthermore, concerns highlighted by medical insurance should be prioritized and their inclusion in NHI implementation processes are essential.

Participants were probed further on their perspectives on reimbursement mechanism that will be utilized under the NHI Fund for PHC services rendered. Majority of participants expressed that a capitation-based approach was the most suitable in comparison to a performance-based model. A capitation-based model is when there is a base fee per patient registered. The base fee will be adjusted according to the health associated risk inherent to the population being services.^20^ The payment for performance (P4P) plan was developed to ensure that quality and positive patient outcomes was rewarded instead of incentivized outputs primarily. Health care professionals are rewarded when predetermined outcomes are efficiently attained.^21^ A study on provider payment methods found that PHC expenditure in low-middle income countries (LMIC’s) remain fragmented and reliant on out-of-pocket payments.^18^ The findings also exhibited that there was high out-of-pocket expenditure on PHC medicines from private pharmacies. This was due to medicine stockouts in public healthcare facilities or provider payment exclusions. This led to inefficient use of medicines and financial burdens on patients.^18^ They advocated that population-based mechanisms such as capitation are the cornerstone for the financing of a patient orientated PHC system. However, these mechanism are rarely utilized in LMIC’s where input budget-based mechanisms such as fee-for-service are prevalent.^18^ Capitation-based reimbursement was the primary mechanism of reimbursement in higher income countries, as it directly links the populations needs to service delivery. A mixed payment mechanism, as mentioned by participants was introduced for specified services to incentivize additional patient health outcomes.^18^ Establishing the correct reimbursement arrangements is critical in driving PHC service delivery and healthcare provider responses to population healthcare needs.^18^ It is also imperative that financing of medicines and the service delivery package is comprehensive to enhance availability and reduce out-of-pocket expenditure. Therefore, there is a pressing need for the South African government to clearly detail reimbursement processes for healthcare providers contracted under the NHI Fund.

## Conclusion

Private medical insurance companies have crucial experience in accrediting providers and billing systems. Participants support the government’s efforts in re-engineering the PHC system with the key benefit being enhanced access to essential healthcare services. In order to achieve this the quality of care received at a primary level should be standardised. It is also imperative that reimbursement frameworks with clearly detailed service provision and accountability guidelines are made available. Policy developers should give careful consideration to the lack of trust and information expressed by role players such as medical scheme representatives to ensure efficient NHI implementation. The findings of this study have highlighted several gaps in the NHI Bill at the current point in time. The lack of these details in the Bill could be supplemented by harnessing the experiences and established systems currently in place within these organizations.
